# The Landscape of Iron Metabolism-Related and Methylated Genes in the Prognosis Prediction of Clear Cell Renal Cell Carcinoma

**DOI:** 10.3389/fonc.2020.00788

**Published:** 2020-05-22

**Authors:** Yanhua Mou, Yao Zhang, Jinchun Wu, Busheng Hu, Chunfang Zhang, Chaojun Duan, Bin Li

**Affiliations:** ^1^Department of Oncology, Xiangya Hospital, Central South University, Changsha, China; ^2^Key Laboratory of Cancer Proteomics of Chinese Ministry of Health, Institute of Medical Sciences, Xiangya Hospital, Central South University, Changsha, China; ^3^State Key Laboratory of Reproductive Medicine and Department of Urology, The First Affiliated Hospital of Nanjing Medical University, Nanjing, China; ^4^Department of Thoracic Surgery, Xiangya Hospital, Central South University, Changsha, China; ^5^National Clinical Research Center for Geriatric Disorders, Xiangya Hospital, Central South University, Changsha, China

**Keywords:** iron metabolism, methylation, ccRCC, DEGs, GSEA, WGCNA

## Abstract

**Background:** Clear cell renal cell carcinoma (ccRCC) is characteristics of resistance to chemotherapy and radiotherapy. The prognosis of ccRCC was dismay with immense diversity. Iron metabolism disturbance is a common phenomenon in ccRCC. The purpose of our study is to identify and validate the candidate prognostic gene signature of iron metabolism and methylation closely related to the poor prognosis of ccRCC through comprehensive bioinformatics analysis in The Cancer Genome Atlas (TCGA) and the Gene Expression Omnibus (GEO) databases.

**Methods:** The prognostic iron metabolism-related genes were screened according to the overlapping differentially expressed genes (DEGs) from the TCGA database. We built a prognostic model using risk score method to predict OS, each ccRCC patient's risk score was calculated, and the resulting score can divide these patients into two categories according to the cut-point risk score. The prognostic significance of the hub genes was further evaluated with the Kaplan-Meier (KM) survival and Receiver Operating Characteristic (ROC) curve analysis. Univariate and multivariate Cox regression analysis was implemented to evaluate the impact of each variable on OS. Furthermore, the prediction power of the 25 gene signatures has been validated using an independent ccRCC cohort from the GEO database. The Gene Set Enrichment Analysis (GSEA) identified the characteristics of hub related oncogenes. Finally, we utilize Weighted Gene Co-expression Network Analysis (WGCNA) to investigate the co-expression network based on these DEGs.

**Results:** In this study, we identified and validated 25 iron metabolism-related and methylated genes as the prognostic signatures, which differentiated ccRCC patients into high and low risk subgroups. The KM analysis showed that the survival rate of the high-risk patients was significantly lower than that of the low-risk patients. The risk score calculated with 25 gene signatures could largely predict OS and DFS for 1, 3, and 5 years in patients with ccRCC.

**Conclusions:** Taken together, we identified the key iron metabolism-related and methylated genes for ccRCC through a comprehensive bioinformatics analysis. This study provides a reliable and robust gene signature for the prognostic predictor of ccRCC patients and maybe provides a promising treatment strategy for this lethal disease.

## Introduction

The incidence of RCC has been increasing in the recent decades, which accounts for 3% of all adult malignancies and ranks the 13th most common malignancy diagnosed worldwide annually. RCC originates from renal epithelial cells (1), and nearly 70–80% cases of RCC is ccRCC pathologic subtype (2). Late diagnosis is a major obstacle to improving ccRCC outcomes, with the fact of about 33% of ccRCC patients initially diagnosed as advanced stage and 40% of those will eventually have distant metastasis. Metastatic ccRCC is associated with poor prognosis, with survival rates at 1, 3, and 5 years, respectively of 50, 30%, and less than 11.2% (3). Surgery remains the mainstay of primary treatment for this disease, but extra therapeutic strategies were required for advanced and metastatic cases. Unfortunately, radiotherapy and chemotherapy exhibit ineffective function, neither immunotherapy has a feeble role in the management of these tumors largely due to its special molecular characteristics involving the regulation of at least one metabolic pathway, which is mediated by iron, oxygen, nutrient, and energy stimulation.

Iron is an essential element in basic biological processes, which contributes to a multitude of crucial physiologic processes. Due to rapid uncontrolled cell proliferation, cancer cells are more dependent on iron metabolism, and subsequently more susceptible to iron depletion, a phenomenon called iron addiction (4). Previous studies unraveled that the disorder of iron metabolism is a common phenomenon in most tumors (5–7), and it is involved in the process of tumorigenesis, angiogenesis, invasion, and metastasis (8). Iron overload in cancer cells, causing redox imbalance and generation of excessive reactive oxygen species (ROS) (8), which induce cell death through membrane lipid peroxidation named as ferroptosis (9–11).

Essentially, ccRCC is a metabolic disease (12). About 70% ccRCC patients were characterized by VHL gene mutation, which was widely accepted as a contributor for the pathogenesis of ccRCC. Besides VHL, other common genes involved in the occurrence of ccRCC such as TSC1, TSC2, SDH, FH, MET, and FLCN were also considered as manipulators of the metabolic pathway, which was mediated by iron, oxygen, nutrient or energy stimulation. Accumulated data also indicate that iron accumulation exists in ccRCC, and iron complexes such as ethylenediaminediacetic acid (Fe-EDDA) and nitrilotriacetic acid (Fe-NTA) can induce ccRCC occurrence (7, 13), whereas high levels of serum ferritin can reduce the risk of ccRCC by lowering the serum level of iron (14).

Recently, the fast-growing studies of ferroptosis have flared up the researches on the role of iron on the pathogenesis of ccRCC and gradually attracted clinicians to the topic of the correlation between iron metabolism-related genes and the prognosis of RCC (15). As vital molecules related with iron-accumulation, ABCG2 expression together with FTH1 mRNA and TFR1 level have been displayed as the negative predictor of ccRCC prognosis (16–18). Conversely, another iron metabolism-related gene ALDH6A1 was reported of its expression positively relating to OS rate in ccRCC patients. Therefore, here we integrated 25 iron metabolism-related and methylated genes and build a predictor model for ccRCC prognosis based on the data from conducting comprehensive bioinformatics analysis, aiming to explore the possible application of ferroptosis-induction in the treatment of this catastrophic disease.

**Figure 1:**
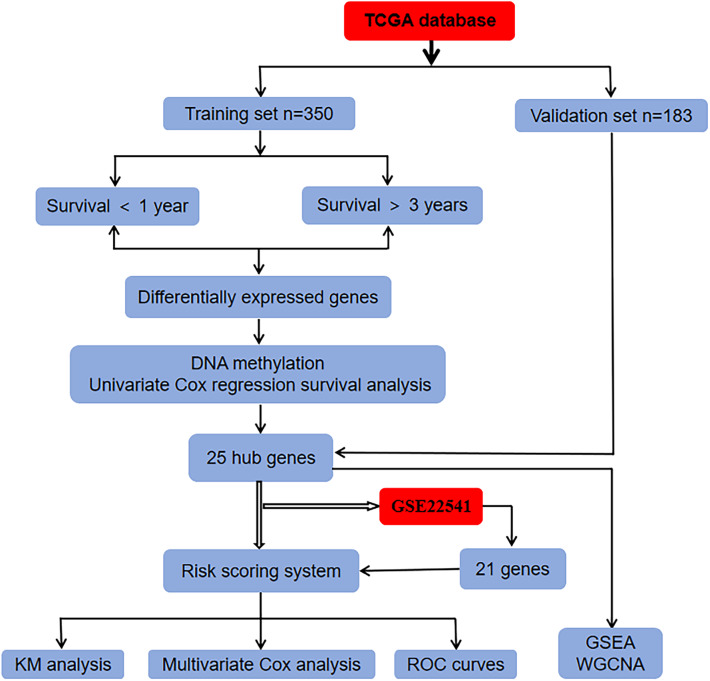
Flowchart of the whole analysis process.

In the present study, we downloaded RNA sequencing, clinical information, and methylation dataset from the TCGA database and identified DEGs by overlapping the candidates through integrated bioinformatics analysis. A risk score system of ccRCC was constructed and validated in the TCGA and GEO databases. Then, the prognostic value of the hub genes was further evaluated via ROC and KM survival analysis (Figure 1). In conclusion, the present study shows that the 25-gene signature could be used as an innovatively independent predictor of prognosis in ccRCC and maybe provide rational therapeutics for personal treatment in ccRCC via ferroptosis-induction.

## Materials and Methods

### Data Source

Sixteen iron metabolism-related gene sets (GO_IRON_ION_BINDING, GO_IRON_ION_IMPORT, GO_IRON_ION_TRANSPORT, GO_RESPONSE_TO_IRON_ION, HEME_BIOSYNTHETIC_PROCESS,HEME_METABOLIC_PROCESS, REACTOME_IRON_UPTAKE_AND_TRANSPORT, HALLMARK_HEME_METABOLISM,MODULE_540,GO_HEME_METABOLIC_PROCESS,GO_2_IRON_2_SULFUR_CLUSTER_BINDING, GO_4_IRON_4_SULFUR_CLUSTER_BINDING, GO_IRON_COORDINATION_ENTITY_TRANSPORT, GO_IRON_ION_HOMEOSTASIS,GO_CELLULAR_IRON_ION_HOMEOSTASIS, GO_HEME_BIOSYNTHETIC_PROCESS) were first extracted from the Molecular Signatures Database v5.1 (MSigDB) (http://software.broadinstitute.org/gsea/index.jsp) (19, 20). The iron metabolism-related gene sets contained a total of 506 genes after removing overlapping genes.

### Patient Data

The TCGA (https://portal.gdc.cancer.gov/) is a free database of largescale cancer genome project which provides clinic and pathological information of 33 types of cancer for researchers (21, 22). This study is based on data from public resources and therefore does not require the approval of the Ethics Committee. The RNA sequencing, clinical information, and DNA methylation data of ccRCC were downloaded from the TCGA database. Five hundred and thirty-three patients were randomly assigned to the training set (*n* = 350) and validation set (*n* = 183). The GEO (http://www.ncbi.nlm.nih.gov/geo/) database, a comprehensive library of gene expression, is a free public database (23–26). Using “ccRCC” as the search term, relevant data sets were screened from the GEO database. The GSE22541 database contains 24 patients with clinical information and corresponding gene expression data.

### Identification of Hub Genes

We firstly screened candidate prognostic genes from the training set. Five hundred and six iron metabolism-related genes were screened out only 409 genes in the TCGA database. The 350 ccRCC samples were applied for identifying prognosis-related genes in the training set. The cut-off point was set as the associated *P*-values <0.05 and log fold change (FC) >0.5, the DEGs between patients with ccRCC surviving for <1 year and those surviving for more than 3 years were analyzed. Furthermore, the volcano plot of all genes in the training set was drawn with a ggplot 2 R package (version 2.2.1, https:/cran.r-project.org/web/Packages/ggplot 2) (27). Then, we detected the methylation status of CpG sites in different gene locus. In order to screen genes related to prognosis, we used survival software package in R to carry out univariate Cox proportional hazard regression. The selected gene *p* < 0.05.

### Risk Score System Establishment

The polygenic risk score is a method used to assess the risk of an individual suffering from a disease. A risk score system for ccRCC patients was constructed in view of the selected hub genes. The prognostic risk score could be constructed on account of a linear combination of the selected genes expression level (exp) multiplied by regression coefficients (β) derived from the univariate cox regression model. Each patient's risk score is calculated as the sum of each gene score; the formula is as follows:

Risk score = expr ${}_{\text{gene}1^{*}}$ β _gene1_ + expr ${}_{\text{gene}2^{*}}$ β _gene2_ + expr ${}_{\text{gene}3^{*}}$ β_gene3_…….expr ${}_{\text{gene}{\text{n}}^{*}}$ β _genen_

Based on this formula, the risk score of each ccRCC patient was calculated. According to the median risk score, the patients were divided into high- and low-risk groups.

### Statistical Analysis

KM curve analysis was performed and examined by the Log-rank test between the low- and high-risk groups. The ROC survival analysis was conducted to compare the predictive accuracy of ccRCC patients in view of the gene signature′ risk score. A *P* < 0.05 was considered to indicate a statistically as the significant difference.

### Multivariate Cox Analysis and Stratified Analysis

Multivariate Cox proportional hazards regression analysis was used to assess whether DEGs could be used as an independent prognostic factor of patient survival in the training, validation, and GSE22541 datasets. Using stratified analysis to analyze the difference of clinical factors between the high-risk and low-risk groups.

### Gene Set Enrichment Analysis

Gene Set Enrichment Analysis (GSEA), which can be acquired from the Broad Institute Gene Set Enrichment Analysis website (http://software.broadinstitute.org/gsea/index.jsp), is a computational method used to analyze gene expression (28, 29). In order to elucidate the relationship between 25 hub gene expression and tumor-related gene signatures, an enrichment analysis of biological processes in high-risk groups were conducted using GSEA tools.

### Weighted Gene Co-expression Network Analysis

To explore the regulatory network of hub genes, we use weighted gene co-expression network analysis (WGCNA) to analyze the fused network, which can describe the gene expression profiles of related patterns (30, 31). The “WGCNA” package in the R language was employed to evaluate the significance and create the co-expression network of the hub genes and their module membership, the characteristics of the network are visualized by using Cytoscape3.6.1 (http://www.cytoscape.org/) (32, 33).

## Results

### Identification of Hub Genes

**Figure 2:**
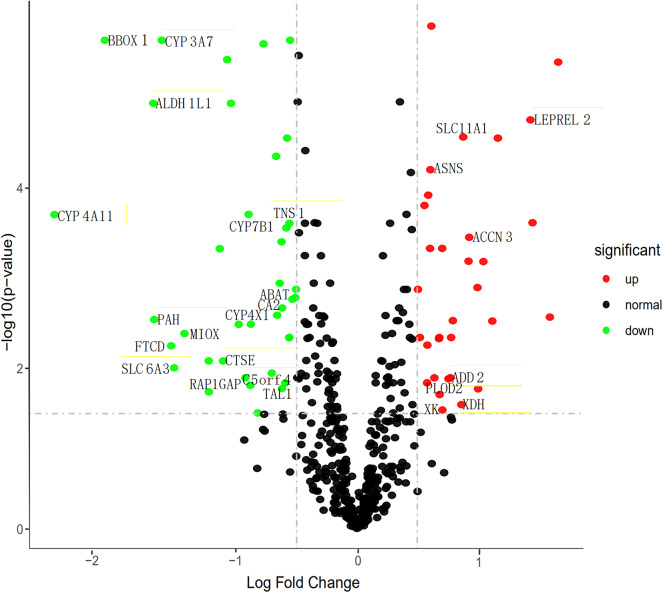
Volcano map describes the distribution of downregulated and upregulated DEGs. Green, black, and red respectively represent low, equal, and high expression of genes in the corresponding group. X-axis: fold change; Y-axis: -log10 FDR value.

To find the vital genes in the progress of ccRCC through gene expression and methylation analysis, firstly, we studied the global transcriptome differences between patients who survived <1 year and more than 3 years using training set in the TCGA database. According to DEGs selection criteria |logFC| >0.5, FDR <0.05,the results showed that 79 genes (35 significantly downregulated and 44 significantly upregulated genes) differentially expressed between the two groups, using the “limma” package of R software. The volcano plot of the upregulated and downregulated DEGs is displayed in Figure 2. Subsequently, the DNA methylation levels of gene promoters in these patients were compared. 450K methylation data of ccRCC patients were downloaded from the TCGA database to obtain the expression and methylation profiles. Then, the correlation between gene expression and CpG site methylation of 79 DEGs was analyzed after all data were standardized via the Z-score method. As a result, 25 differentially expressed genes were obtained.

We then performed a univariate Cox regression analysis on the 25 DEGs in the training set. Under the cut-off threshold of Cox *P* < 0.05, we found these 25 DEGs were considered as survival-related genes, which may have significant prognostic value for ccRCC, named hub genes. The general information of these 25 hub genes is displayed in Table 1.

**Table 1:** The 25 filtered genes through gene expression and methylation analyses.

**Gene**	**FDR**	**Hazard ratio**
SLC11A1	0.0076	1.467069983
TAL1	0.001220897	0.802344011
PLOD2	0.001203141	1.405641565
RAP1GAP	0.007322931	0.836694499
LEPREL2	1.25E-06	1.215710073
ASNS	3.38E-06	1.625119932
ALDH1L1	3.80E-07	0.847414505
ADD2	0.004730745	1.149522208
BBOX1	2.46E-08	0.876370533
CTSE	0.002600651	0.91766366
ABAT	0.000368623	0.760918584
XK	0.014649957	1.166012549
SLC6A3	0.003327568	0.942137564
CYP7B1	4.18E-05	0.693588566
CYP4A11	1.43E-05	0.905753589
CA2	0.000160434	0.735698919
XDH	0.012187999	1.077382059
TNS1	2.20E-05	0.659715961
FTCD	0.001666799	0.926978896
MIOX	0.000983171	0.911296705
CYP4X1	0.000462289	0.81770677
ACCN3	8.27E-05	1.391556503
PAH	0.000578306	0.922750034
C5orf4	0.001180463	0.758878498
CYP3A7	1.40E-08	0.766999378

### Construction and Assessment of Prognostic Risk Scoring System

**Figure 3:**
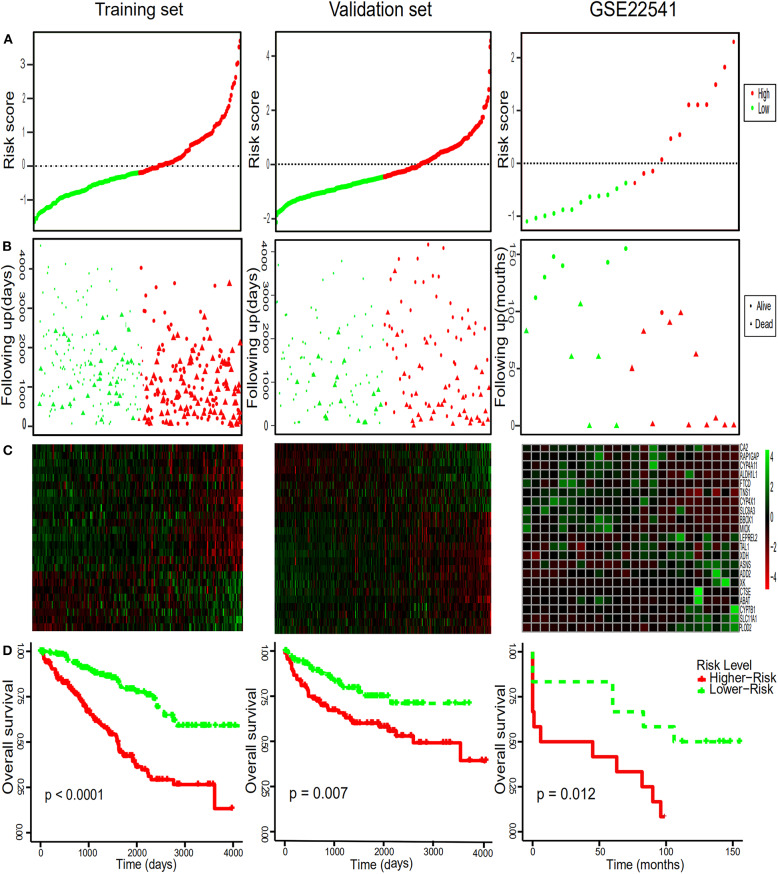
Risk score distribution, survival time analysis of the patients, the heatmaps of the hub genes and Kaplan-Meier analysis. **(A)** The 25-gene signature risk score distribution in the TCGA and GEO datasets. **(B)** The survival status of ccRCC patients from the TCGA and GEO datasets. **(C)** The heatmaps of the 25 hub genes expression profiles in ccRCC. Green indicates a higher expression and red indicates a lower expression. **(D)** Kaplan–Meier survival of OS in TCGA and of DFS in GEO dataset of ccRCC patients according to the median risk score. Red and green separately represent high- and low-risk groups.

To comprehensively study the relationship between these 25 genes and the prognosis of ccRCC, a 25-gene survival risk scoring system based on gene expression level and Cox coefficiency was calculated. In the prognostic model, each patient with ccRCC was classified into high-risk and low-risk groups according to the median risk score value as the cut-off point in the training and validation sets. The prognostic risk score for each patient was calculated and plotted in Figure 3A. Besides, the distribution of survival time (Figure 3B) and corresponding heatmap of hub genes expression level (Figure 3C) in the training and validation sets were also presented.

### Diagnostic Values of Hub Gene

**Figure 4:**
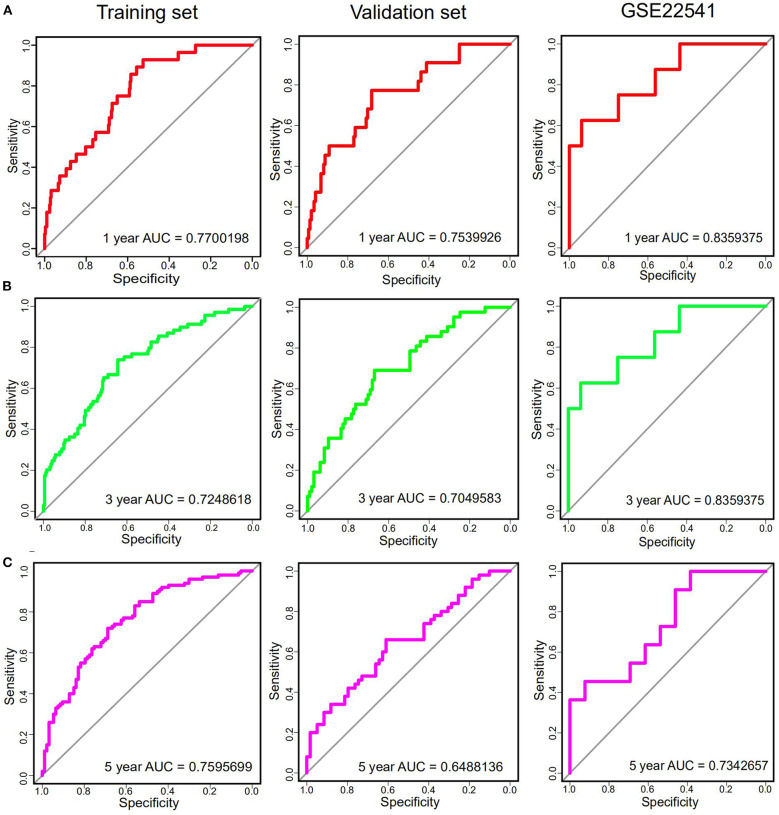
The time-dependent ROC curves analysis indicating the sensitivity and specificity of predicting 1 **(A)**, 3 **(B)**, and 5 **(C)** years of survival according to the 25-gene signature based on risk score in training, Validation and GSE22541 datasets.

In order to further assess the integrated effects of the low and high-risk score groups on the prognosis, we performed the KM curve analysis of OS in the TCGA data (Figure 3D). Patients in high risk scoring group had significantly shorter survival ratio in comparison with low-risk score group in the training set (*p* < 0.0001). For the validation set, consistent with our previous description, patients in the low-risk groups presented a significantly longer OS time than patients in the high-risk groups *(p* = 0.007). Furthermore, The 25-gene signature achieved the ROC values (AUC) of 0.7700198 (90%CI 0.6887261, 0.8513134), 0.7248618 (95%CI 0.6553988, 0.7943248), and 0.7595699 (95%CI 0.6922502, 0.8268896) respectively for 1, 3, and 5-year OS in the training set (Figure 4). Similarly, the 25-gene signature could predict the 1, 3, and 5-year OS of patients with ccRCC in a great measure, the area under the AUC was 0.7539926 (95%CI 0.6442222, 0.8637631), 0.7049583(95%CI 0.6123979, 0.7975187), and 0.6488136 (95%CI 0.5450598, 0.7525673) in the validation set, which implicates that the 25-gene signature shows an efficacious performance for OS prediction. By and large, these results indicated that the 25-gene signature for predicting prognosis of ccRCC patients is robust.

### Further Validation of the 25-Gene Signature Using an Independent Cohort

Notably, in order to verify the reliability of the 25-gene signature in predicting the prognosis of ccRCC patients, another independent ccRCC cohort from the GEO database was used for further validation. Among the 25 DEGs, only 21 DEGs (The absence gene: ACCN3, PAH, C5orf4, CYP3A7) were in the GSE22541 dataset. KM survival analysis exhibited that the ccRCC patients with high risk score were significantly shorter DFS compared to those in the low risk groups (*P* = 0.012) (Figure 3). ROC curve analysis for the 21-gene prognostic signature was 0.8359375(95%CI 0.6571055,1), 0.8359375(95%CI 0.6571055,1), and 0.7342657(95%CI 0.5288579,0.9396735) at 1, 3, and 5 years of DFS in GSE22541 dataset, implying that the 21-gene signature is reliable and valid for DFS prediction across datasets and platforms (Figure 4). The corresponding risk scores, heatmap of hub genes expression level, and the distribution of survival time in the GSE22541 dataset were also presented.

### Multivariate Cox Regression Analysis of the Core Gene

In order to determine whether the hub genes may be as an independent variable correlated with poor prognosis in patients with ccRCC, we used multivariate Cox regression models in both TCGA and GEO cohorts. We employed a multivariate Cox proportional regression analysis including 25-gene risk score, age, gender, histologic grade, pathologic T, pathologic N, pathologic M and pathologic stage to authenticate that the 25-gene risk classifier can serve as an independent and reliable determinant of OS in patients with ccRCC in the training and validation cohorts respectively (*p* < 0.001, *p* = 0.003) (Table 2). Subsequently, Multivariate Cox regression, containing gender, histologic grade, pathologic T, pathologic N, pathologic M and risk score, was also performed in GSE22541 dataset, confirming that the 21-gene signature was an independently predicting prognosis indicator in ccRCC patients (*p* = 0.045). Relevant analysis exhibited that four clinical factors (histologic grade, pathologic T, pathologic M, pathologic stage) in high-risk and low-risk groups were of significant differences (Table 3).

**Table 2:** Multivariate Cox regression analysis of the gene signature was performed in ccRCC patients.

**Database**	**Variables**	**Univariate analysis**		**Multivariate analysis**	
		**HR (95%CI)**	***P–*value**	**HR (95%CI)**	***P–*value**
Training	Age	1.016 (0.995–1.038)	0.144	1.024 (1.001–1.048)	0.037
	Gender	1.153 (0.682–1.948)	0.596	0.999 (0.568–1.759)	0.998
	Histologic grade	1.893 (1.342–2.669)	<0.001	0.787 (0.492–1.258)	0.317
	Pathologic T	1.785 (1.358–2.345)	<0.001	1.129 (0.608–2.098)	0.701
	Pathologic N	4.615 (2.072–10.28)	<0.001	1.038 (0.428–2.516)	0.934
	Pathologic M	3.611 (2.070–6.299)	<0.001	1.801 (0.671–4.835)	0.243
	Pathologic stage	1.697 (1.360–2.119)	<0.001	1.119 (0.600–2.087)	0.725
	Risk score	1.227 (1.162–1.294)	<0.001	1.203 (1.126–1.285)	<0.001
Validation	Age	1.026 (0.997–1.055)	0.077	1.048 (1.006–1.091)	0.023
	Gender	0.976 (0.489–1.951)	0.946	1.461 (0.646–3.305)	0.363
	Histologic grade	2.694 (1.660–4.372)	<0.001	2.015 (1.081–3.756)	0.027
	Pathologic T	1.882 (1.203–2.943)	0.006	0.755 (0.326–1.752)	0.513
	Pathologic N	1.587 (0.482–5.229)	0.447	2.763 (0.707–10.805)	0.144
	Pathologic M	4.515 (2.223–9.172)	<0.001	4.071 (1.006–16.468)	0.049
	Pathologic stage	2.062 (1.409–3.018)	<0.001	1.228 (0.464–3.250)	0.679
	Risk score	1.145 (1.068–1.228)	<0.001	1.138 (1.045–1.239)	0.003
GSE22541	Gender	1.299 (0.493–3.423)	0.597	0.544 (0.176–1.686)	0.292
	Histologic grade	0.898 (0.292–2.760)	0.851	0.347 (0.086–1.407)	0.138
	Pathologic T	1.219 (0.668–2.226)	0.519	0.996 (0.487–2.040)	0.992
	Pathologic N	3.397 (1.265–9.125)	0.015	15.71 (2.195–112.5)	0.006
	Pathologic M	20.1 (3.443–117.42)	<0.001	75.0 (2.902–1921.2)	0.009
	Risk score	1.145 (1.068–1.228)	0.001	1.203 (1.004–1.440)	0.045

**Table 3:** Results of the stratified analysis under high-/low-risk groups.

**Clinical factor**	***P*-value (training set)**	***P*-value (validation set)**
Age	0.415	0.959
Gender	0.027	0.205
Histologic grade	2.325e-07	0.024
Pathologic T	1.755e-05	0.001
Pathologic N	1.563e-04	0.460
Pathologic M	0.026	0.027
Pathologic stage	2.704e-05	0.002

### GSEA

**Figure 5:**
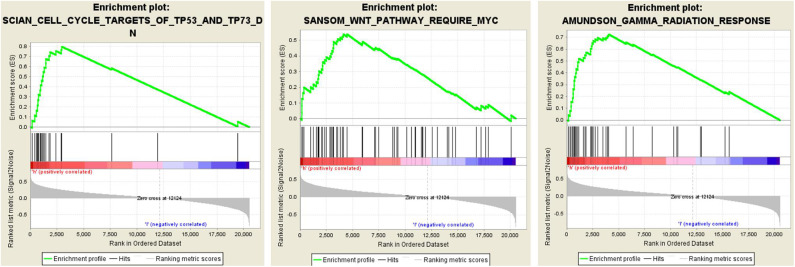
Twenty-five gene signatures correlated enrichment gene analysis with GSEA, only three of the most common functional gene sets were listed, all of which were considered to be significantly enriched in high-risk groups when compared with the low-risk patients.

To elucidate the potential influence of hub genes expression on the expression profile of ccRCC. The enrichment analysis of the biological process of gene ontology was conducted by using Gene Set Enrichment Analysis v4.0.0 software obtained from the Broad Institute (www.broadinstitute.org/gsea). GSEA analysis of the merged dataset revealed that higher risk score was enriched in genes regulating SANSOM_WNT_PATHWAY_REQUIRE_MYC (NES = 1.8464239, *p* = 0.001988072), SCIAN_CELL_CYCLE_TARGETS_OF_TP53_AND_TP73_DN (NES =1.7889026, *p* =0.017408123), and AMUNDSON_GAMMA_RADIATION_RESPONSE (NES = 1.7706424, *p* =0.036538463) (Figure 5). It was illustrated that cancer cells with the higher risk scores of iron metabolism-related signature could be closely related to the cell cycle phase. Generally, the above results show that our combined data are qualified to reflect expression profiles of cancer samples and biological characteristics.

### WGCNA

**Figure 6:**
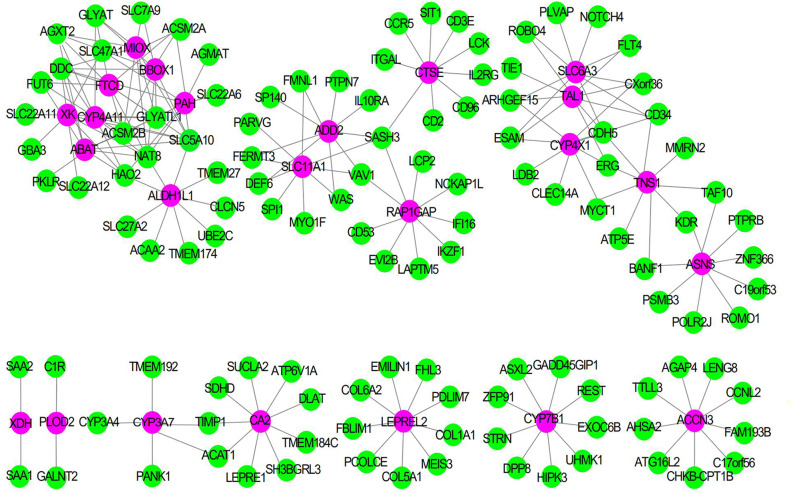
The Co-expression network of 25 gene signatures. Rose red nodes show the key genes, and green nodes are genes which co-expressed with key genes.

To explore the synergistic gene modules of 25 core genes, we employed the WGCNA to examine the co-expressed genes using the WGCNA package in the R environment. WGCNA network construction showed that 25 modules possessed corresponding expression patterns. A total of 119 target genes were discovered to be co-expressed with these hub genes in the co-expression network. The co-expression network of the 25 genes is visualized by WGCNA in Figure 6.

## Discussion

The ccRCC is a heterogeneous disease in light of molecular characteristics, diverse morphologies, metabolic pathways, therapeutic response and clinical outcomes. At present, although with some limitations of low accuracy and specificity, it was still widely accepted that size, grade, vessel invasion and morphological characteristics of tumor, as well as patient performance were considered as significant prognostic indictors for ccRCC patients. In order to improve prognostic prediction, this study is to identify and validate the candidate prognostic gene signature of iron metabolism and methylation closely related to the poor prognosis of ccRCC through comprehensive bioinformatics analysis in the two public databases.

With the development of epigenetics and metabolomics, the identification of biomarkers in many cancers has been extensively studied. We analyzed the gene expression profiles of the TCGA and GEO databases in order to identify the gene signature that can predict the poor prognosis of ccRCC patients. Twenty-five core genes related to DNA methylation and the prognosis of ccRCC were screened out from 493 iron metabolism-related genes. We constructed 25 core gene signature that can be used to stratify patients into high and low risk groups. The prognostic value of the key genes was further evaluated with ROC and KM survival analysis in the training, validation and GSE25441 datasets. More importantly, the risk score generated from 25 hub genes can be used as a novel independent prognostic indicator, as it can availably predict the OS and DFS for 1, 3, and 5 years in patients with ccRCC. Furthermore, we also applied GSEA and WGCNA analysis to explore the biological processes associated with these hub genes, which laid the foundation for further basic researches on the effect of 25 hub genes on the pathogenesis of ccRCC and then providing possible targets for its treatment.

Iron is involved in processes related to DNA replication and maintaining genomic integrity (including DNA repair). DNA synthesis thus directly depends on components of iron–sulfur cluster biogenesis from mitochondria and the cytosol. A dinuclear iron site is essential for the catalytic activity of both constitutive and p53-inducible ribonucleotide reductase, which catalyzes the rate-limiting step in DNA synthesis, the reductive conversion of ribonucleotides (NDPs) to deoxyribonucleotides (dNDPs). Iron deficient cells accumulate in G1 phase of cell cycle, which is consistent with the key role of iron in DNA synthesis. The proteins that control cell cycle are also regulated by iron levels (34). Tachpyridine, iron chelator, can induce G2 arrest and selectively sensitize cancer cells to ionizing radiation, suggesting that iron chelators may function in anticancer therapy as radioenhancing agents (35).

Iron is widely involved in a great deal of physiologic processes, such as DNA replication, chromatin remodeling, DNA repair, mitochondrial metabolism and the cellular stress response. As the same as the metabolism of other essential metals, iron metabolism includes absorption, transport and utilization. SLC11A1 is a well-known promoter for iron transport, which play a significant role in regulating iron homeostasis. ACCN3 can also regulate the transport of iron ion. Of these 25 hub genes, PLOD2, LEPREL2, BBOX1, CYP7B1, CYP4A11, XDH, MIOX, CYP4X1, PAH, C5orf4, CYP3A7, XDH, ABAT, and SLC6A3 are all involved in the regulation of iron ion homeostasis. RAP1GAP, ASNS, ALDH1L1, ADD2, CTSE, XK, CA2, TAL1, TNS1, and FTCD affect iron metabolism by regulating the process of heme metabolism.

Among these 25 hub genes, some were reported to be participated in the process of metastasis and invasion of ccRCC. Kurozumi et al. demonstrated that PLOD2 encodes a kind of collagen lysine hydroxylation enzyme, whose aberrant expression promotes extracellular matrix (ECM) stiffening, leading to the enhancement of cancer cell invasion and migration (36). ADD2 is a kind of membrane skeleton protein belonging to the adducin family, which plays a pivotal role in regulating metastasis of ccRCC. Li et al. collaborated that MIR-218 inhibited the metastasis and invasion of endometrial cancer by inhibiting ADD2 (37). Tensin 1 (TNS1), a component of specialized fibrillar adhesions, which is a molecular bridge linking the actin cytoskeleton, signal transduction and extracellular matrix has been implicated in the regulation of cell migration in ccRCC. Zhou et al. reported that the level of TNS1 gene and protein in CRC was higher than that in normal tissues and cells. TNS1 signal transduction promotes the invasion and proliferation of CRC cells (38). Rap1GAP, an important tumor suppressor, impedes the invasion and migration of cancer cell through the downregulation of Rap1. Kim et al. showed that the decrease of Rap1GAP expression due to the promoter hypermethylation can stimulate the invasion of RCC cells (39). ALDH1L1, cytosolic 10-formyltetrahydrofolate dehydrogenase, inhibited the invasion and migration of cancer cells via a specific folate-dependent mechanism (40). Previously published studies have now shown that ALDH1L1 is universally and strongly downregulated in malignant tumors by its promoter methylation (41). Consistent with the previous study, Chen et al. confirmed that the decrease of ALDH1L1 expression in hepatocellular carcinoma cell (HCC) is closely correlated with poor prognosis of HCC patients (42).

The research confirmed that the high expression of SLC6A3 is correlated with short recurrence-free survival (RFS) in ccRCC patients with post-surgery (43). XDH, an iron-containing protein, has an enormous influence on the development and transformation of various cancers. Liu et al. reported that the survival rate of gastric cancer patients with higher XDH expression was significantly lower than that of lower XDH expression (44). CYP4X1, monooxygenases, encodes a member of the cytochrome P450 superfamily of enzymes. Wang et al. suggested that CYP4X1 inhibition can prolong the survival rate of glioma (45). In accordance with these, our study has shown that iron metabolism-related genes are correlated with the prognosis of ccRCC, but their actual effects and potential mechanisms remain unclear.

Some studies have authenticated that iron metabolism related-genes SLC11A1, ASNS, SLC6A3, and FTCD may be involved in the regulation of tumorgenesis (46–49). But the value of these genes in the occurrence of ccRCC and its underlying mechanism needs to be further explored.

**Figure 7:**
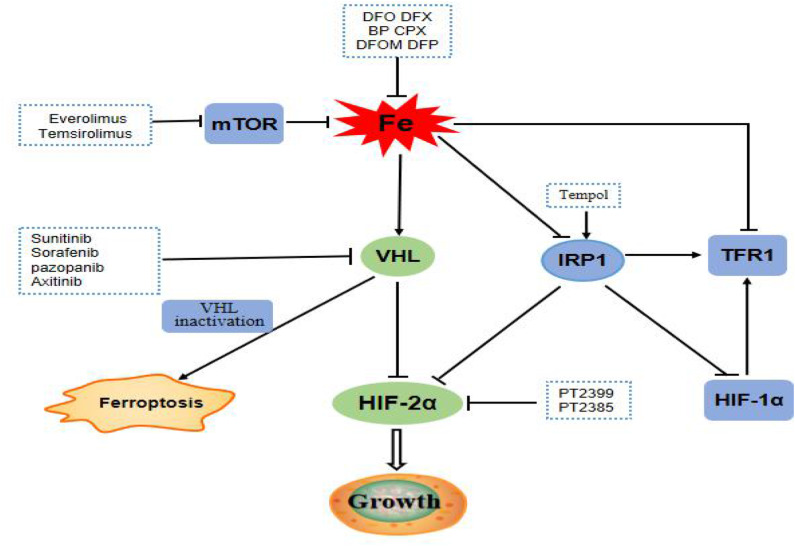
mTOR pathway is a regulator of iron metabolism, targeted drugs everolimus and temsirolimus target the mTOR pathway to treat ccRCC patients. The VHL/HIF-α axis, which plays a central role in the carcinogenesis of ccRCC, is the main regulator target of iron metabolism. Targeting drugs such as sunitinib, sorafenib, pazopanib, and axitinib targeted at VHL gene pathway. Inactivation of VHL increases the sensitivity of ccRCC to ferroptosis. IRP1 can bind to the iron reaction element of HIF-2α mRNA and inhibits its translation. Tempol, an IRP1-activated drug, inhibits HIF-2α and HIF-1α protein levels. PT2399 and PT2385 are inhibitors of HIF-2α.

ccRCC harbor the traits of primary resistance to chemotherapy and radiation therapy, mainly due to the dysregulation of vonhippel-lindau- hypoxia-inducible factor- vascular endothelial growth factor (VHL-HIF-VEGF) pathway resulting from VHL gene mutation (Figure 7). Interestingly, the VHL-HIF-α axis participated in the government of iron metabolism inside ccRCC cells (50, 51). VHL inactivation increases the sensitivity of ccRCC cell to ferroptosis, which is an iron-dependent form of programmed necrosis mainly triggered by extra-mitochondrial lipid peroxidation arising from an iron-dependent ROS accretion (52). The survival, proliferation, and invasion ability of ccRCC cells were highly dependent on the elevated level of HIF-α and free iron concentration (50). Mounting evidences showed that the efficacy of drug targeting the VHL might be influenced by the turbulence of iron metabolism somewhat deriving from the abnormality of its related genes expression. Therefore, it was plausible that iron metabolism mediated ccRCC drug resistance to target therapy and the related genes possibly forecast the prognosis of ccRCC. Different from other tumors, the natural resistance of ccRCC is one of the important factors leading to the poor prognosis of patients with ccRCC. Our study also confirmed that iron metabolism-related genes might be able to assess the prognosis of ccRCC patients.

The PI3K/Akt/mTOR pathway was abnormally activated in ccRCC cells, and therefore targeting this pathway, whether alone or together with other drugs, has great potential in the remedy of ccRCC (53). Intriguingly, one of the momentous regulators of iron metabolism is the mTOR pathway, which mostly fine-tunes the rate of iron importation required from the needs of cells. As a result, everolimus, a mTOR inhibitor can increase the levels of TTP and iron with decrease TFR1 levels (54). Hence, the aberrance of gene balancing iron metabolism will lessen the role of mTOR inhibitor and consequently lead to treatment failure. These results further confirmed that iron metabolism-related genes might influence the prognosis of ccRCC by interceding drug resistance.

Over the past few years, the use of VEGF-targeting agents or VEGF followed by mTOR blockage has been a prevailing treatment paradigm for ccRCC. Currently, sunitinib and sorafenib targeting VHL-HIF-α pathways were approved as the first-line treatment of ccRCC. But preliminary failure to these drugs was not rare and second resistance will eventually occur, which represents a major hurdle for the achievement of cures. Everolimus, as the only drug targeting mTOR in ccRCC, is the first approved second-line drug of ccRCC, and also prone to loss of efficacy in ccRCC. Therefore, it is conceivable that the genes engaging in the iron metabolism maybe provide an alternative target for ccRCC patients. Future studies may further explore the effects of targeted drugs, chemotherapeutic drugs, and even radiotherapy on iron metabolism-related proteins or iron levels in patients with ccRCC. Up to now, the treatment strategy of targeting iron mainly focuses on the cellular iron exhaustion and repletion. The iron deprivation has been achieved by using iron-chelating agents including DFX and DFO, which show the safety and effectiveness of clinical value for patients. The yeast extract can inhibit the proliferation of RCC cells by regulating iron metabolism (55). These results provide the possibility for the combination of iron chelation or yeast extract with targeted therapy which warrant more researches.

The advantages of our research are as follows. First of all, we developed an iron metabolism-related signature for the first time and confirmed that it was closely associated the OS and DFS of ccRCC patients in two databases (TCGA and GEO). Secondly, the screened prognostic predictors were significantly correlated with DNA methylation, which plays an indispensable role in the occurrence and development of ccRCC. Lastly, according to the median risk score, the prognosis of ccRCC patients can be evaluated independently with high sensitivity and specificity. However, our study has a few limitations. First, the sample size of the GSE22541 was small, with only 24 ccRCC samples. Secondly, there are only 21 hub genes in the GSE22541 dataset, not all 25 core genes have been verified. Thirdly, the GSE22541 dataset only provides DFS data, lack of OS data, so we only calculated the risk factors of gene signature associated with DFS in GSE22541 dataset. Fourth, the TCGA and GEO databases may have platform differences, data heterogeneity, and sample size differences. Large samples are needed to validate the findings of this study and following functional experiments of these genes in the pathogenesis of ccRCC were necessary for consolidating the key role of ferroptosis-inducer in treating this deadly disease.

## Conclusion

In this work, based on a comprehensive bioinformatics analysis from TCGA and GEO databases, we developed an iron metabolism-related and methylated gene signature for predicting ccRCC prognosis and demonstrated for the first time that it was closely related to the 1, 3, and 5 years- OS and DFS in ccRCC patients. These findings open up a new horizon that iron metabolism may take part in the pathogenesis and the invasion behavior of ccRCC. And it also possibly provides a promising prospect in developing ferroptosis-induction therapeutic strategy, especially for delaying or reversing the resistance of ccRCC to the anti-angiogenesis or mTOR inhibition treatment.

## Data Availability Statement

The datasets analyzed in this study can be found in The Cancer Genome Atlas (https://portal.gdc.cancer.gov/).

## Author Contributions

BL, CD, and CZ designed the article. BL and CD modified the manuscript. YM and YZ drafted the manuscript and were responsible for the acquisition of data. JW and BH contributed to the literature search. YM performed the statistical analysis. All authors read and approved the manuscript and agree to be accountable for all aspects of the research in ensuring that the accuracy or integrity of any part of the work are appropriately investigated and resolved.

## Conflict of Interest

The authors declare that the research was conducted in the absence of any commercial or financial relationships that could be construed as a potential conflict of interest.
